# Ending the organ trade: an ethical assessment of regulatory possibilities

**DOI:** 10.1007/s40592-025-00232-7

**Published:** 2025-03-01

**Authors:** Andreas Albertsen

**Affiliations:** Aarhus University, Aarhus, Denmark

**Keywords:** Organ trafficking, Organ markets, Controversial markets, Organ trade, Organ donation

## Abstract

While the trade of human organs are illegal and widely condemned, a black market flourishes. Estimates indicate that 10% of kidney transplants from living donors involve illegal payments to the kidney seller. This paper presents a typology for approaches aimed at curtailing the black market in human organs. The policies are evaluated from two perspectives: their ethical permissibility and their expected efficiency in ending and minimizing the trade in human organs. To end or minimize organ trading, we must reduce the organ shortage in order to reduce demand for organs, alleviate poverty to reduce the supply of organs, and disincentivize brokers and medical facilitators through a concerted effort to reduce the profit rate of the international organ trade.

## Introduction

The shortage of organs for transplants inflicts immense suffering. Across the globe, people die while waiting for an organ. Given this dire situation, it is of little surprise that some of those in desperate need for an organ are willing to employ drastic measures. Some people waiting for a kidney transplantation opt to buy one instead of waiting for an organ that may not become available until it is too late. In doing so, kidney buyers circumvent international statutes and local legislation in a desperate attempt to prolong their lives. Intermediaries connect them with those who are willing to give up one of their kidneys for money.

For both sides of this transaction, the opportunities for pursuing such a path are not difficult to find. A quick Google search reveals several web pages offering to buy people’s kidneys. Sellkidney.com was set up to facilitate public debate about kidney sales. It informs those who stumble upon the site that buying a kidney is illegal. The comments on the page, however, are flooded with people offering to buy and sell kidneys (“Sell Kidney – Kidney Market” 2017). The Facebook page “I want to sell my kidney” provides a chilling view into human desperation, as those wanting to sell and those wishing to buy find each other through posts and comments. A BBC inquiry into British citizens buying kidneys in Pakistan found that they did so at prices ranging between $50,000 and $60,000 (Evans 2017; Aagaard 2020; Interpol 2021). The desperate situation of the buyers is matched by the desperation of those willing to sell. At Tsunami Nagar, one of the refugee camps created in India for people relocated after the 2004 tsunami, so many have opted to sell their organs that the camp is nicknamed Kidneyville (Carney 2011, 68). Reports from Pakistan (Moazam et al. 2009), India (Goyal 2002), the Philippines (Cohen 2015, 281), and elsewhere confirm that sellers are in plentiful supply. Whichever route is taken, buying an organ involves the circumvention of the near-universal ban on organ sales.

News outlets regularly report on the organ trade. Whether giving voice to desperate buyers, impoverished sellers, or those who make the organ business tick, they deliver fascinating insights into the workings of the organ trade (Ginzel et al. 2012; Vora 2016). Over the last two decades, a number of books have also discussed various aspects of the trade in organs (Carney 2011; Cohen 2015; Dickenson 2008; Lundin 2015; Territo and Matteson 2011; Waldby and Mitchell 2006; Columb 2020). This paper seeks to advance the current thinking regarding how the organ trade can be minimized. Its premise is that it would be better if fewer sales were made. While there is an interesting and important discussion to be had about the permissibility of organ sales (Albertsen 2020, 2023a, 2023b, 2024a, 2024b; Beard et al. 2013; Radcliffe-Richards et al. 1998; Richards 2012; Rippon 2014, 2017; Semrau 2014, 2023; Semrau and Matas 2022; Taylor 2005, 2007, 2009, 2014; Thaysen and Sønderholm, 2024; Wilkinson 2003), this article side-steps this discussion to present and evaluate different policies aimed at achieving the curtailment of organ trade. It thus seems to inform what we should do, if we adopt the current dominant legal framework were organ trade is illegal. It provides a typology of different solutions and analyzes their efficacy and ethical permissibility. The hope is to provide a philosophical discussion which is informed by our empirical knowledge regarding the organ trade.

## The organ market: What is it?

The illegal status of organ trading makes it notoriously difficult to assess the extent of the market. The buying and selling of organs is even harder to detect than other illegal activities, because only some forms of the activity are illegal. We cannot deduce from the very fact that a person has received a transplant that something illegal has taken place. One is permitted to pay to receive a transplant if the organ is donated rather than sold. Even the fact that a transplant happened in another country is not a giveaway. This is the case because a direct donation to a stranger is not illegal either. It is the payment to the donor that is illegal, and this creates problems of assessing the size and spread of the organ trade.

 Serious efforts have been undertaken to overcome these difficulties and offer a clear picture of the extent of this activity. In 2007, Shimazono conducted the most comprehensive study available. It estimates that commercial organ transplants amount to 5% of all organ transplants (Shimazono 2007). The most frequently traded organ is the kidney. Estimates suggest that kidneys account for 75% of traded organs. 10% of the kidney transplantations conducted worldwide are commercial—as a headline in a newspaper might put it, an organ is sold every hour. That kidneys are the most frequently sold organ is of little surprise for two reasons. First, kidneys are the organ for which demand is highest. Kidneys are also one of the organs that can be removed from living sellers/donors, which—all else being equal—may increase the incentive for selling. It also removes coordination problems because it is possible to remove the organ after the recipient has been flown in, whereas organs from deceased sources create difficulties because organ quality deteriorates rather quickly (the specific ischemia time varies between organs). The organ market is big business. A report by Global Financial Integrity estimated that the illegal organ trade yields a profit between $600 million and $1.2 billion every year. The report notes that this places it tenth on the list of the most profitable illegal activities (Haken 2011). The organ market flourishes across the globe, also according to a new review (Alnour et al. 2022). The majority of organs are bought in countries that have little capacity and/or willingness to address the situation (Pascalev et al. 2016a, 34).

It is necessary to have a clear understanding of who the participants in the global organ market are (Scheper‐Hughes 2000, 2001, 2004). While the illegal nature of the market makes such an assessment difficult, it is important to undertake because some understanding of who buys, sells, and operates on the organ market is fundamental to assessing the likely effect of various proposals to deter such activity. *Organ recipients* are those who need an organ transplant and pay to receive one. *Organ brokers* receive the money from the organ recipients and facilitate relevant contacts and the required setup for the transplant to take place (Cohen 2015, 281–283; Columb 2020, 48–49; Lundin 2015; Meyer 2006). *Organ sellers* are those who agree to exchange their organs for money, and *Medical facilitators* are those who, often employed by the organ brokers, use their medical skills to remove and transplant organs. This includes transplant surgeons as well as those who perform various medical tests on the organ recipients.

What we know about the typical kidney seller is gained from various surveys and interviews conducted among former kidney sellers. Sellers are typically poor and in vulnerable positions (Columb, 2020, 5,73; Lundin 2015; Pascalev et al. 2016a, 35). They seldom receive the total sum they were promised (Goyal 2002; Lundin 2015, 27; Pascalev et al. 2016a, 36), and post-transplant medical follow-up is more often pledged than received. Their post-transplant outcomes are not encouraging, as they often report that their health has deteriorated and their economic situations have not improved (Cohen 2015; Goyal 2002; Moazam et al. 2009; Moniruzzaman 2012; Budiani‐Saberi and Delmonico 2008). Given what we know about kidney sellers, their socio-economic position, and the risks they are willing to undertake, it seems reasonable to assume that kidney sellers are likely to be desperate. Regarding kidney buyers, there is not a lot of available information. The few available studies find that the medical outcomes from purchased kidneys are generally worse than the outcomes from comparative non-commercial transplantations (Cohen 2015, 178–280; Al Rahbi and Al Salmi 2017). In terms of who the buyers are there is little available knowledge (Cohen 2015, 277) we gain little reliable knowledge from the survey. Based on the prices listed in the literature reporting on the organ trade, we can infer that they must be quite well off (Lundin 2015, 3)- it is common in the literature to refer to them as rich (Pascalev et. al. 2016a, 31). We can furthermore readily assume that they are desperate enough to pay considerable amounts to avoid finding out what awaits them if they remain on the waiting list. Lundin writes that a common thread in her conversations with Swedish buyers were 'long illness and desperation' (Lundin 2015), also be people who are inadequately covered by their national health insurance (Columb 2020, 23). Finally, brokers and medical facilitators are a broad group, and one which we know little about. Regarding Medical Facilitators. It seems fair to assume that the vast majority of them know that what they are doing is illegal. It is difficult to know whether their motives are monetary or nobler, but as reports suggest that facilitating the global organ trade is a lucrative business for many of those involved, we may reasonably postulate that the monetary gains are an important part of their motivation.But there there is little knowledge about the transplant professionals (Pascalev et al. 2016a, 44). Part of the complexity of the role and knowledge of medical professionals is that the transplants are often carried out within the remits of the official system (Columb 2020, 58, Lundin 2015, 58). It is often (deliberately) unclear whether the patient pays for the service of receiving a transplant (which is legal) or the organ (which is not legal) (F. Ambagtsheer et al. 2016). One reason for this is surely that participants in the trade seek to ensure that everything looks legal(Manzano et al. 2014; Columb 2020). Some confronted doctors claim to be unaware of being part of the trade (Lundin 2015, 74), for others it is not considered their job to check the sources or validate consent forms (Columb 2020). Brookers are often former organ sellers (Columb 2020, 50), and it seems fair to assume that the money is a large part of their motivation for entering the business (often part-time). It is a varied group, some work across nations, in groups - others locally and alone (Pascalev et al. 2016a, 39; Columb 2020, 47-50). Several researchers have stressed that the oft told horror stories relating it to organized crime are not an adequate picture (Columb 2020), and stressed that the focus on trafficking may be counterproductive (Columb 2015, 2020; Columb et al. 2017).

## Legal aspects

This section briefly presents the legal status of organ trading and highlights the stance of important organisations. The first law in the United States which regulated the procurement of organs, The Uniform Anatomic Gift Act, was passed in 1968 and made no mention of trade in organs (Chapman 1982, 399; Hansmann 1989, 57–58; Robinson 1999, 1026). Several real-world events led this policy stance to be considered inadequate (Altman 1994, 170; Robinson 1999, 1026). When deregistered Virginia physician H. Barry Jacobs formed an organisation called the International Kidney Exchange, with the purpose of buying kidneys from living sellers, it sparked immense controversy (Fox and Swazey 1992, 65; Sullivan 1983; Robinson 1999, 1036). As existing legislation could not stop Jacobs from setting up his business, the National Organ Transplant Act was passed in 1984. It effectively banned the trade of organs in the United States (Robinson 1999, 1029).

 The WHO moved in a similar direction. In 1987, the World Health Assembly initiated a process which would draft the first WHO Guiding Principles on Transplantation. These were endorsed by the assembly in 1991 (WHO 2010). These principles called for all countries to enact a ban on organ sales (Altman 1994, 172–76; WHO 1989, 1991). This discussion was also shaped by real-world events, such as people posting newspaper ads proposing to buy or sell kidneys (Altman 1994, 170) and the sale of kidneys by Turkish farmers in London (LA Times 1989). The stance of the WHO has since been reaffirmed, and in 2010 the World Health Assembly endorsed an updated version of the guiding principles. The position of the WHO is supported by a number of international documents and declarations. Most influential has been the Declaration of Istanbul, issued in 2008 at a summit convened by the Transplantation Society and the International Society of Nephrology (Participants in the International Summit on Transplant Tourism and Organ Trafficking Convened by The Transplantation Society and International Society of Nephrology in Istanbul, Turkey, April 30 through May 2, 2008 2008). Over 100 organizations across the world have since signed the declaration. In 2013, 70 members of the Declaration of Istanbul Custodian Group met in Doha, Qatar to discuss the Istanbul declaration on its fifth anniversary. They passed the Doha Communique, which re-affirms the stance against organ trafficking, highlights a number of initiatives taken against it, and stresses the importance of registers, transparency, and cross-border cooperation (The Declaration of Istanbul Custodian Group 2013). A stance later confirmed by the 2018 version of the Declaration of Istanbul (Martin et al. 2019). It is frequently suggested that this policy document was important to anti-trafficking laws passed in Pakistan and the Philippines (White et al. 2014). Additionally, the WHO Transplant Observatory was established in 2005 to increase actual knowledge regarding transplantation data worldwide (GODT 2023). The Observatory follows trends in legislation and transplant activity. On the EU level, we have also seen several recent reports drafted on the issue, most notably a report by The Council of Europe, UN and the European Parliament (Bos, European Parliament, and Directorate-General for External Policies of the Union 2015). The European Parliament(European Parliament 2008) has also passed legislation aimed at minimising and deterring the organ trade. A fuller picture of the legislation is provided by others elsewhere (Ambagtsheer and Weimar 2016; Columb 2020, 3-6).

### A typology of initiatives to decrease organ trafficking

This section presents a typology of the different measures we could undertake to minimise or deter the organ trade. While the employed categories are connected, it is important to distinguish them from each other. The purpose of what follows is to show how different measures fall into different categories and to assess their likely effects.

Markets bring together those willing to buy with those willing to sell. Black markets as an economic phenomenon express how the willingness to buy and sell a given commodity is larger than the deterrent provided by criminalisation. Initially, we can distinguish between initiatives which seek to decrease the demand for black market organs and those which seek to reduce supply. We can also, however, distinguish initiatives by whom they target. Each initiative aims to promote some change in behaviour from one or more of those who are currently engaged in the black market. This provides us with the following categories:

**Table:** 

Target/channel	Supply-side initiatives	Demand-side initiatives
Organ sellers		
Organ recipients		
Organ brokers		
Medical facilitators		

Ultimately, this paper will evaluate various initiatives regarding how efficient we can expect them to be. Efficiency will be understood as the ability of a measure to diminish the organ trade at a comparatively reasonable cost and without erecting significant barriers to legal organ donations.Considerations of fairness will also be raised when relevant.

## Strategies focused on curtailing the supply of black market organs

This section discusses various initiatives aimed at decreasing the supply of black market organs. Many of the strategies discussed are also mentioned in the Hague recommendations (Caulfield et al. 2016; Pascalev et al. 2016b; Holmes et al. 2016) 

### Strategies targeting sellers

#### Poverty reduction

Given that sellers are poor, this is a measure which should be part of the discussion (Columb 2020; Pascalev et al. 2016b; Zutlevics 2001). Those lifted out of poverty would likely not be willing to sell their kidneys, given that desperate economic circumstances are a prominent motivation for organ sellers. In one way, this is likely to be an effective measure. As desperation is widely recognised as an important driver for potential sellers, improvement in living conditions would be an effective deterrent in the sense that it would make it much less attractive to pursue the selling of one’s organs.

#### Information campaigns

This strategy involves attempts to provide information to potential kidney sellers, with the purpose of deterring them from participating in organ trafficking. Informing sellers about the dangers involved and the dubious nature of the brokers they are dealing with could be part of such a strategy. Pursuing this route faces several important obstacles, however. Providing adequate information to people who may have very low education levels is in itself difficult. Another important aspect is the desperate situation of potential sellers. If selling a kidney is considered one of the best options available, information about the fact that it is illegal and risky is unlikely to be enough to deter people. This is not to claim that those choosing to sell their organs always understand what they are agreeing to. It is well-documented that the use of misinformation about the nature of organ donation is a frequent tactic among organ brokers to lure potential sellers into participating. Taken together with the discussion on poverty reduction above, it seems reasonable to suggest that information campaigns would only be effective if they worked in tandem with initiatives to improve people’s overall prospects. Given what has been stated about the desperation of sellers, prosecution of seller should be considered unfair.

### Strategies targeting brokers

Another strategy would be to curtail the market via the options available to would-be sellers through a concerted effort to deter people from acting as brokers.

#### Seizing profits

Concerted efforts to prosecute and punish organ brokers is one obvious alternative (seizing criminal profits would make the market less lucrative). The main obstacle for such a strategy is the current legal setup in many of the countries where the organ market thrives. These countries are likely to have a difficult time enforcing such measures. Given that a number of international organisations are concerned with the trade in organs, and that the organ trade has strong links with other criminal activities, perhaps there is a role here for international organisations.

#### Registering

It is problematic if inadequate testing and transparency allow purchased organs to flow into the regular transplantation system. For this reason, it has been suggested that there is a greater need for transparency (Cherry 2005). If purchased organs cannot enter the ordinary transplantation system, this limits the demand for organs in a very specific way by limiting how lucrative the organ trade is for those participating in it. Such a strategy however, requires those who today participates in illicit transaction to report these.

### Strategies targeting medical facilitators

What could be done to deter those who employ their medical expertise in the service of the organ trade?

#### Information campaigns

One option is deterrents aimed at medical facilitators, such as more information about possible sanctions and the dubious nature of the business to which they are contributing. This is unlikely to be effective. It is reasonable to think that those facilitating the trafficking of organs know what they are doing. In that case, they are likely to be strongly motivated by the financial gains to be made through this practice and not likely to be talked out of it.

#### Concerted efforts to prosecute and punish medical facilitators

Here the effort is to punish those who facilitate the organ trade. This could be through fines, loss of licenses, and even prison. This seems a better strategy in terms of what might work as a deterrent. Yet this is only the case if the laws are enforced, which is currently unlikely. Nevertheless, given the monetary motivation among facilitators, it makes sense to try to make it less profitable and more risky to participate in the trade. The strategy however, relies on effective implementation and this is unlikely. Furthermore, the complex nature of the trade also makes criminal prosecution difficult (Ambagtsheer, 2020; Holmes et al. 2016; Pascalev 2016). It is also a recurrent worry that focussing too much on criminalization and prosecution may have adverse effects (Columb, 2020; Ambagtsheer, 2020; Ambagtsheer and Weimar 2016). 

## Strategies focused on curtailing demand for black market organs

There is also another branch of alternatives, namely those focused on the demand side of the organ trade. These strategies focus on reducing demand in one way or another.

### Strategies targeting buyers

#### Decreasing the demand for organs as such

One way of reducing the demand for black market organs would be to decrease the demand for organs in general. After all, if fewer people needed organs for transplants, the shortage would decrease and with it the waiting lists and the pressure to turn to commercial transplantation. However, this option is more a theoretical possibility than a viable strategy: historically, new technologies and new options for treatment have increased rather than decreased the need for organs. For that reason, the shortage of organs is persistent and is likely to be so for the foreseeable future. Recall also that the current shortage—those listed as currently waiting for an organ—in effect underrepresents the real need for organs, as people die while waiting or are taken off the waiting list because they become too sick to undergo the transplantation procedure.

#### Decreasing demand for black market organs

Another possible strategy would be to focus on increasing the number of organs available from other sources. The rationale behind such an approach is that if we increase the supply of organs procured without relying on current black market practices, we would probably be able to crowd out the black market. It seems plausible that few would pursue the black market route if they had a viable alternative. For example, a survey among residents in Oman who turned to commercial transplantation found that the lack of a properly functioning donation system within their country was an important reason for their choice of commercial transplantation (Al Rahbi and Al Salmi 2017). As people turn to the black market to supply them with an organ because they lack viable alternatives, providing such alternatives is likely to decrease the demand for black market organs.

#### Information as a deterrent

In the context of public health, an oft-employed strategy is to deter people from undertaking specific actions by providing information. We can imagine information campaigns targeting people who could be interested in utilising transplant tourism. Such campaigns or doctor initiated coversations could discuss possible adverse consequences, and the dangers involved in the commercial kidney trade on the part of the organ buyers (Caulfield et al. 2016; Pascalev 2016b). Both studies and meta-studies conducted on those undergoing commercial transplantation find that patients face severe risk, higher than those undergoing regular procedures. This includes not only short-term risks of complications, but studies also show commercial transplantation to be an independent risk factor for long-term kidney allograft loss (Al Rahbi and Al Salmi 2017). The Declaration of Istanbul website already features information for those considering purchasing a kidney. It informs them about unfavorable conditions for organ sellers, and the medical dangers for organ buyers (Declaration of Istanbul Custodian Group 2014). This suggests a slightly different strategy, aimed at evoking moral doubts among potential buyers. While such strategies are almost always uncontroversial, we also have reason to harbour doubts regarding their efficiency. Telling people that it is dangerous to buy a transplant organ is only likely to be helpful if they have an alternative to doing so. Given the desperate situation of potential buyers, they are likely to dismiss such warnings, and we may have reason to fear that any ethical doubts they may have about buying a kidney from someone living in abject poverty will be set aside for the same reasons.

#### End public reimbursement for illegal transplants

As documented in detail in Cohen’s work on medical tourism, it is increasingly common that health insurers will cover medical treatment in other countries (Cohen 2015). Until recently, several EU countries would reimburse the costs of illegal transplants (Bos, European Parliament, and Directorate-General for External Policies of the Union 2015, 66). The problem with such practices is two-fold. They create an incentive for health insurance companies to (at least to some degree) aid in the trade in human organs, as this is often cheaper than traditional transplants. Secondly, such practices lower the costs to individuals of obtaining a kidney from the black market. This makes such illegal practices available to more people. Raising the price would be one way of lowering demand. While the prices referred to earlier were those offered to organ sellers, these are only a fraction of the expenses (and profits) that the organ buyers (or their insurers) have to provide.

#### End health-based subsidies for recipients of illegal transplants

Another measure which serves to increase the costs borne by organ recipients who opt for the black market would be to require them to pay for treatment for complications and/or for the drugs that they need as a consequence of their transplant. As with the insurance-based deterrents mentioned above, these measures would serve as a deterrent for would-be organ buyers, and they would, in the end, make sure that they do not pass the cost onto others. It is, of course, an empirical matter whether such a policy is implementable, but some countries, including the United States, already allow for this (Cohen 2015). Would such a policy be unfair towards those who are affected by it? It is not the case that they would be denied treatment; they are only denied free treatment. Is it unreasonable that those who seek a health benefit in a way that enriches criminal organizations are asked to pay their own way if this endeavor gives rise to complications? On the other hand, the desperate situation of the organ buyers speaks against punitive measures - and in general, punishment through withholding health services may seem add odds with other values.

#### Legal action against organ buyers

Finally, there is also the possibility of introducing even harsher sanctions against those who buy an organ and thereby contribute to the organ trade. On the assumption that they are committing a wrong in buying an organ, it would seem plausible to argue that such a wrong should not go unpunished. Two considerations count against this: the first is that if they face prosecution, the willingness of organ recipients to cooperate with authorities is likely to be diminished. This could mean that valuable insights into the organ trade would not be brought to the fore (on the other hand, perhaps the threat of punishment and the possibility of reducing that punishment through providing information is a solution worth considering). The second consideration against punishing organ buyers springs from considerations of fairness. While it is difficult to say when people are acting freely in choosing what to do, it should be taken into account that those opting to buy an organ are under considerable duress, and thereby to at least some extent could be excused, if not justified, for doing as they do.

#### Decreasing the demand for organs from black market sources through the creation of viable alternatives

An important aspect of minimising the organ trade is to increase the supply of organs from legal sources (Meyer 2006; Aronowitz and Isitman, 2013). As there is a vast literature on this, I will only briefly describe some of the available measures in this category:

- Creating a deceased donor registry. As many countries have not established a registry, and thus do not have a working donation system, inhabitants of those countries have little choice other than to turn to commercial transplantation.
- Initiatives to increase the donor rate. Even in countries with a functioning donation system, it is well known that donor rates vary between countries. For this reason, numerous European countries have adopted opt-out policies, where people are considered donors unless they register an objection to this. It should also be stressed that the above-mentioned proposals of establishing regulated markets in organs or providing economic incentives for donations should also be considered as one measure available here. Another proposal are priority rules, which gives priority to previous donors or registered donors (Albertsen 2017, 2023; Steinberg 2024a, b).
- Initiatives to limit family refusals. In many countries, families may veto the wish of the deceased to donate. This potentially lowers the actual donation rate and is one problem to address to increase the number of organs procured.
- Initiatives to increase the utilisation rate (the number of organs utilised from each deceased donor). There is variation among countries regarding their success in using the organs from those who are both willing to donate and medically fit to do so (i.e. die under the proper conditions from a donation perspective, do not have illnesses which complicate transplantation, etc.).
- The creation of a system for related and non-related living donors. Especially in the context of kidneys, this is important, as post-transplant survival rates remain higher for kidneys from living sources.

#### Mandatory reporting

Organ transplantations are not small procedures. As already mentioned, people receiving a transplanted organ need follow-up care and treatment. As this treatment, including receiving immunosuppressive drugs, is a continuous process, it is unlikely that the transplant recipient will undergo it in the same country where he received his transplant, under the supervision of the medical facilitators who made the transplant possible in the first place. Rather, this process is likely to be undertaken in the organ buyer’s home country. This raises the possibility that a duty to report could be enacted (Manzano et al. 2014). There are two immediate possibilities for how to enact such a rule. One option is to require any doctor or hospital which is asked to supply immunosuppressive drugs to look into where this transplantation was received. In most cases, this could easily be clarified through the local organ allocation authorities. But, in other cases, where people who have been listed on the organ waiting list for months suddenly turn up having had a transplant but are unable to give a coherent story about where they received it or provide any real documentation, then the system should conduct an investigation. Depending on the legal framework enacted, this investigation may not be for the primary purpose of collecting evidence against the organ recipient, but rather because it could be useful in prosecuting other participants in the organ trade. Reporting practices can be supported by developed indicators for medical professionals (Jong and Ambagtsheer, 2016).

## Conclusion

The paper has provided a framework for differentiating different measures to limit the organ trade, as well as a tentative discussion of how we should evaluate these measures. Any attempt to limit the organ trade must employ a wide range of measures, including measures which address the causes of the organ trade. The general line of thought developed here is that to end or minimise organ trading, we must reduce the organ shortage to reduce demand for organs, alleviate poverty to reduce the supply of organs, and disincentivise brokers and medical facilitators through a concerted effort to reduce the profit rate of the international organ trade.

## Data Availability

No datasets were generated or analysed during the current study.
